# Hypercrosslinked porous polyporphyrin by metal-free protocol: characterization, uptake performance, and heterogeneous catalysis

**DOI:** 10.1080/15685551.2016.1259831

**Published:** 2016-11-30

**Authors:** Li-Juan Feng, Min Wang, Zhi-Yong Sun, Yun Hu, Zhen-Tao Deng

**Affiliations:** ^a^ Department of Bioengineering, Zunyi Medical College (Zhuhai Compus), Zhuhai, China

**Keywords:** Adsorption, catalysis, hypercrosslinking, porous polymers, porphyrin

## Abstract

Through metal-free protocol, hypercrosslinked porous polyporphyrin with permanent porosity was obatined via the Friedel–Crafts alkylation of tetracarbazolylporphyrin using formaldehyde dimethyl acetal as an external cross-linker. Its chemical structure and porosity was well characterized and confirmed. The BET specific surface area value of **HCP-TCPP** is 1050 m^2^ g^−1^ and related dominant pore size is centered at 0.63 nm. The adsorption amount of methanol by **HCP-TCPP** is high up to 800 mg g^−1^ (about 25.0 mmol g^−1^) at its saturated vapor pressure, which is higher than that of toluene (600 mg g^−1^, 6.5 mmol g^−1^). Further study indicates that polymer **HCP-TCPP**, possessing the high BET specific surface area and total pore volume, exhibits good hydrogen uptake of 3.44 wt % (77 K) and high carbon dioxide uptake of 41.1 wt % (298 K) at 18.0 bar. Besides, the obtained porous polymer can also be used as an effective heterogeneous catalyst for the Knoevenagel condensation between various aldehydes and malononitrile.

## 1. Introduction

As one of intrinsic nanoporous polymers, hypercrosslinked porous polymer represent a type of porous organic networks with high specific surface area and good thermal/chemical stability, which have been used for the adsorption of gas and organic vapors, the removal of organic compounds from water,[1] and the heterogeneous catalyst of organic transformation.[2] Friedel–Crafts alkylation is a commonly used reaction for preparation of hypercrosslinked porous polymers. Recently, a facile synthetic method to microporous polymers has been developed by Tan’s group, which is a simple one-step Friedel–Crafts alkylation of aromatic monomers using formaldehyde dimethyl acetal (FDA) as an external cross-linker.[3] Through this approach, various hypercrosslinked porous heterocyclic polymers [4,5] have been smoothly obtained for gas uptake & separation, and catalyst immobilization. Besides, various hypercrosslinked porous polymers made in metal-free condensation have reported recently,[6,7] which will be of high importance toward future mass applications.

Owing to the large p-conjugated macrocyclic system containing basic pyrrole cavity, porphyrin may bind Lewis acid CO_2_ through strong interaction. Metal ions can also be readily incorporated into the porphyrin center because of the square-planar coordination site. In recent years, porous organic polymers based on porphyrin have drawn great attentions due to the rigid structure and special function of porphyrin.[8–15] It is reported that metalloporphyrin-based porous materials can promote some important chemical transformation such as photosynthesis, oxygen transport, and catalytic oxidations.[8,9,11,12] However, porphyrin-based porous organic polymers without built-in metal sites in the skeleton have been reported rarely until now.[16,17] Compared with metalloporphyrin based porous materials, the porous polymer based on free-base porphyrin obtained with lower cost can display special properties and function derived from the basic pyrrole containing macrocyclic cavity with large p-conjugated system.

Efficient preparation and flexible molecular design of versatile porous polymers have been proven from the smoothly template-free preparation by selection of various building blocks and polymerization reactions.[18,19] Herein, hypercrosslinked porous polyporphyrin with permanent porosity and special functions was prepared by the Friedel–Crafts alkylation of tetracarbazolylporphyrin in metal-free style using FDA as an external cross-linker. Its chemical structure and porosity was well characterized and confirmed. The related gas adsorption capacities under high pressure for hydrogen and carbon dioxide have been investigated and the capabilities for adsorption of solvent vapors such as toluene and methanol have also been explored. Its adsorption performance can be comparable with some conjugated porous polymers we reported before.[20,21] Owing to the basic pyrrole containing macrocyclic cavities, the obtained porous polymer can also be used as a heterogeneous catalyst for the Knoevenagel condensation between various aldehydes and malononitrile.

## 2. Experimental

### 2.1. Materials

Trifluoromethane sulfonic acid (TFMSA), *o*-dichlorobenzene, and FDA were purchased from Alfa Aesar company. All chemicals and reagents were used without further purification unless otherwise stated. Tetrakis(carbazol-9-ylphenyl)porphyrin (TCPP) was prepared according to the reported method.[22] The chemical structures of all monomers were fully confirmed.

### 2.2. Structure characterization and analysis

Solid-state cross polarization magic angle spinning (CP/MAS) NMR spectrum was obtained on a Bruker Avance III 400 NMR spectrometer. The infrared (IR) spectra were obtained from a PerkinElmer Spectrum One Fourier transform infrared (FTIR) spectrometer. Thermogravimetric analysis (TGA) was carried out on a Pyris Diamond thermogravimetric/differential thermal analyzer by heating (10 °C min^−1^) the samples to 900 °C under the nitrogen or air atmosphere. Scanning electron microscopy (SEM) observation was performed using a Hitachi S-4800 microscope without sputter coating. Transmission electron microscopy (TEM) observation was carried out with a FEI Tecnai G2 20S-TWIN microscope at an accelerating voltage of 200 kV. The SEM and TEM samples were prepared by placing a drop of the sonicated suspension of the as-prepared material in ethanol on a silica wafer and lacey support film, respectively, followed by drying under ambient condition.

### 2.3. Porosities studies and adsorption measurements

Nitrogen sorption isotherms were recorded with Micromeritics ASAP 2020 M+C accelerated surface area and porosimetry analyzers at certain temperature (all the samples were degassed at 120 °C for 12 h before measurement). BET specific surface area and micropore surface area were evaluated based on the obtained adsorption–desorption isotherms. The PSD of materials was calculated from the related adsorption branch by the nonlocal density function theory (NLDFT) approach. Total pore volume was calculated from nitrogen adsorption–desorption isotherms at *P*/*P*_0_ = 0.99. The vapor (methanol and toluene) adsorption–desorption isotherms and gas (H_2_ and CO_2_) uptake capacities were measured on an IGA-100B intelligent gravimetric analyzer.

### 2.4. Preparation of HCP-TCPP

TCPP (0.10 g, 0.081 mmol) was dissolved in anhydrous *o*-dichlorobenzene (20 mL), and then FDA (450 µL, 0.3 mmol), catalyst TFMSA (3.0 mmol) were added to the reaction mixture The mixture was stirred for 1 h under nitrogen at room temperature, and then the mixture was heated to 100 °C and kept at the temperature for 12 h. The crude product was filtered off from the hot reaction mixture and carefully washed with water, ethanol, acetone, and chloroform to remove reagents, solvents and possible low molecular weight by-products. After extracted in a Soxhlet extractor with methanol for 24 h, and then with tetrahydrofuran for another 24 h, the desired polymer **HCP-TCPP** (yield: 95%) was collected and dried in vacuum oven at 110 °C overnight.

### 2.5. General procedure for Knoevenagel condensation

To a suspension of **HCP-TCPP** (1 mol% of the substrates) and dioxane (0.2 mL)/water (0.2 mL) in a 5 mL round bottomed flask were added aromatic aldehyde (0.1 mmol) and malononitrile (0.1 mmol). The reaction mixture was stirred at the room temperature. The formation of the products was monitored by TLC. After 5 h, the mixture was filtered through a short silica gel flash column and purified using petroleum ether/dichloromethane as eluent to afford the desired product.

## 3. Results and discussion

**Scheme 1.**
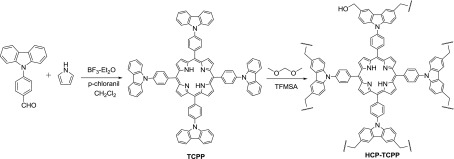
Preparation of hypercrosslinked porous polyporphyrin **HCP-TCPP**.

The synthetic route to porphyrin monomers and corresponding porous polymer is shown in Scheme 1. Condensation of 4-(9-carbazolyl)benzaldehyde with pyrrole was performed under Lindsey conditions to produce the porphyrin derivative **TCPP**,[23,24] in which the Lewis acid catalysis is at high dilution followed by addition of oxidant. Using the **TCPP** as monomers and FDA as the crosslinker, the Friedel–Crafts crosslinking polymerization was promoted smoothly by TFMSA in dry *o*-dichlorobenzene under nitrogen atmosphere at 100 °C. This modified metal-free protocol can furnish the desired hypercrosslinked porphyrin-based porous organic polymer **HCP-TCPP** in high yield. The obtained polymer is stable and insoluble in common solvents as a result of the cross-linking nature.

**Figure 1.**
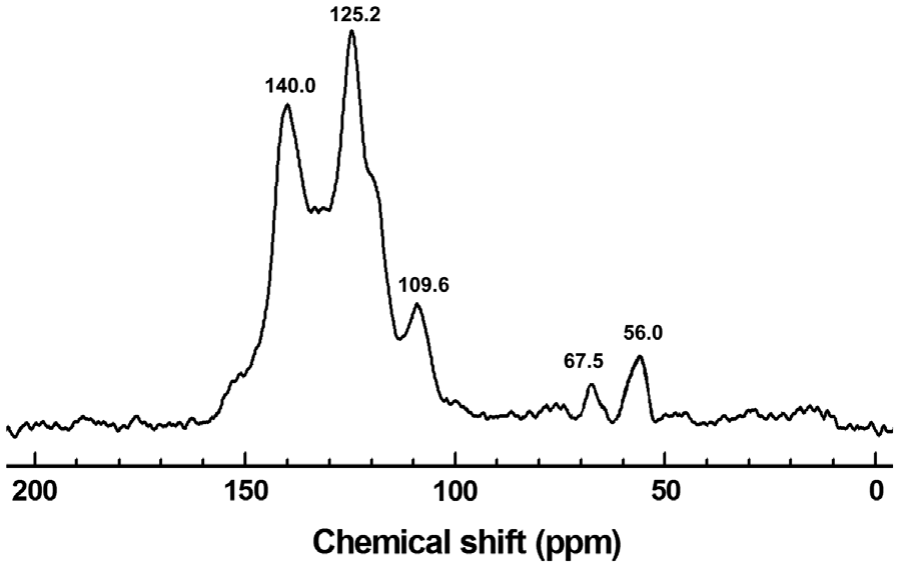
^13^C CP/MAS NMR spectrum of **HCP-TCPP**.

Polymer **HCP-TCPP** is characterized at the molecular level by the solid-state ^13^C CP/MAS NMR spectrum as shown in Figure 1. Generally, the broad peaks at 140–105 ppm are ascribed to the peak of aromatic carbons, in which assignment of the signal resonances related to porphyrin macrocycle and carbazolyphenylene are consistent with reported data. In details, the peak at about 140.0 ppm corresponds to the substituted phenyl carbons binding with nitrogen atom. The high-intensity peak for other substituted phenyl carbons is located at about 125.2 ppm, the signal peak at about 109.6 ppm is ascribed to the unsubstituted phenyl carbons, which are perfectly consistent with the previous work about carbazole-based porous organic polymers.[25–27] Peaks at 68–55 ppm are ascribed to the peak of methylene carbons from the linker FDA. The methylene carbons binding with phenyl, which are derived from FDA reacted with the monomer completely, are located at 56.0 ppm. Meanwhile, the peak at about 67.5 ppm corresponds to methylene carbons binding with oxygen atom, which are ascribed to imperfect reaction of the linker.[28] As we known, there are some methylene carbons that just bind with the monomer partially in the material.

**Figure 2.**
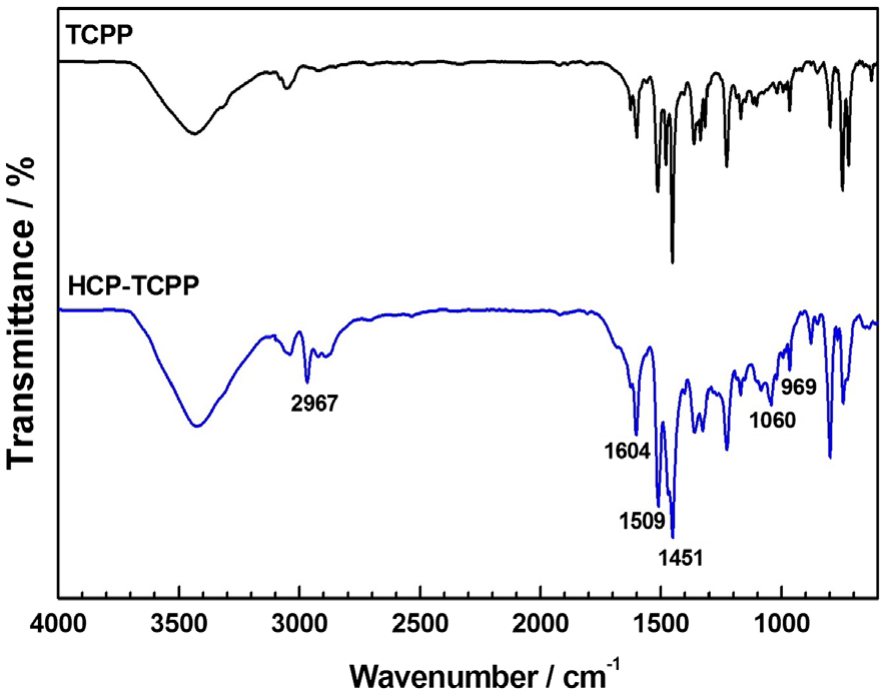
FTIR spectrum of **HCP-TCPP**.

A comparison of the FTIR spectra of monomer and polymer is shown in Figure 2, in which signal peak at about 969 cm^−1^ in the spectra of both monomer and polymer is ascribed to N–H in-plane bending vibration of the pyrrole group. The peaks at about 1604, 1509, and 1451 cm^−1^ are attributed to aromatic ring skeleton vibrations, which were consistent with the structure of monomer and polymer. Besides, as for the spectrum of prepared polymer, we found that the broad absorption peaks at about 3422 cm^−1^ (O–H band stretching vibrations), the weak absorption peaks at about 2967 cm^−1^ (C–H band stretching vibrations) and the peaks at about 1060 cm^−1^ (C–O stretching vibrations) indicate the structure of –CH_2_– and a handful of tail end group such as –CH_2_OH and –CH_2_OCH_3_ in the hypercrosslinked network.[28]

**Figure 3.**
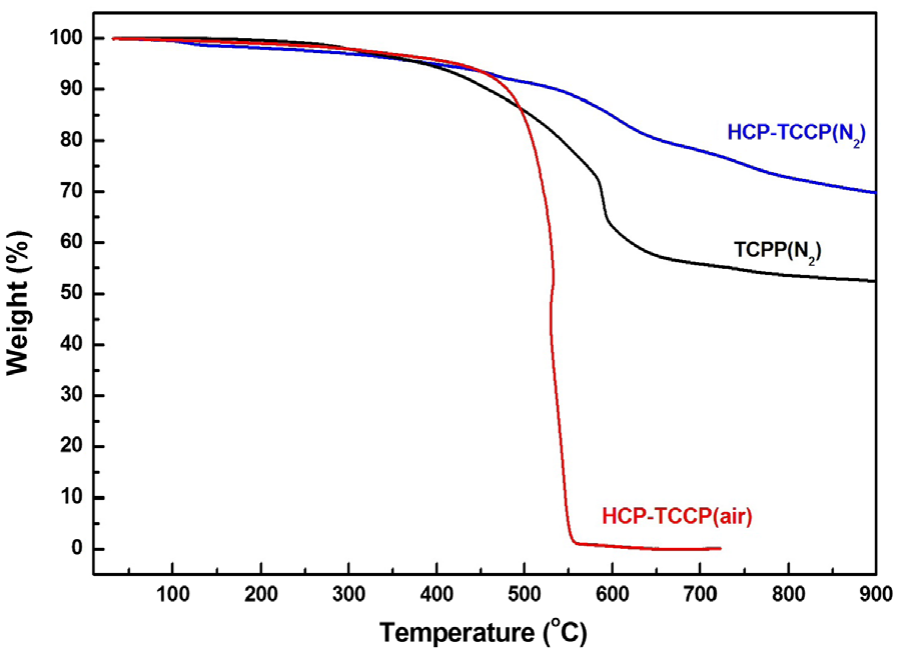
TGA plots of **TCPP** and **HCP-TCPP**.

**Figure 4.**
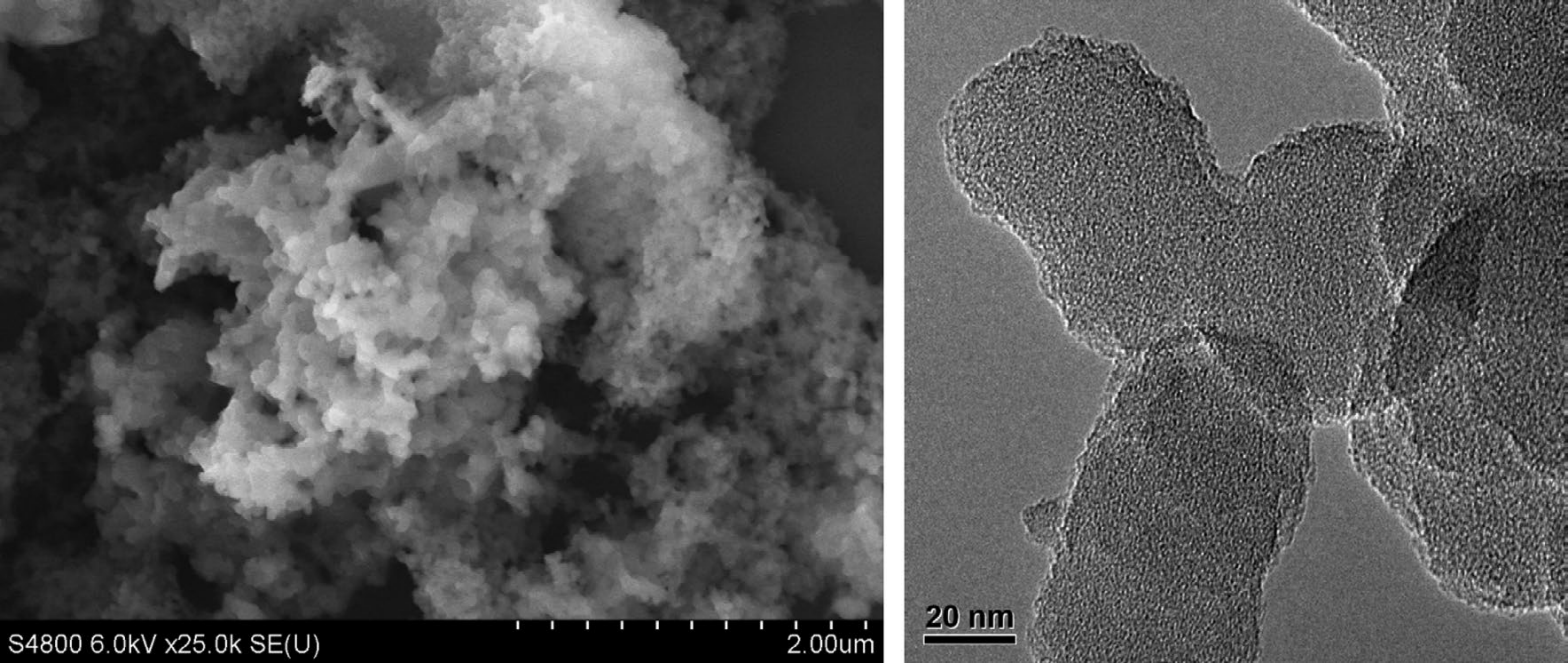
SEM (left) and TEM (right) images of **HCP-TCPP**.

The thermal stability of the polymer was characterized by TGA (Figure 3). Five percent of mass loss was observed at about 420 °C in the air, even similar to that under nitrogen. When the temperature rises to 800 °C, there is less than 30% of mass loss under nitrogen. It has no evidence for distinct glass transition for these polymers below the thermal decomposition temperature due to the nature of their cross-linking structures. For monomer **TCPP**, it appears 35% mass loss at around 635 °C, and the cross-linked polymer **HCP-TCPP** retains the residual masses of 71.8% at 800 °C. From comparing the thermogravimetric results of the network and corresponding monomer, it can be inferred that the formation of hypercrosslinked network take place. The SEM and TEM images of the obtained polymer are shown in Figure 4. Material morphology was observed to be solid submicrometer particles with different size between 70 and 150 nm.

**Figure 5.**
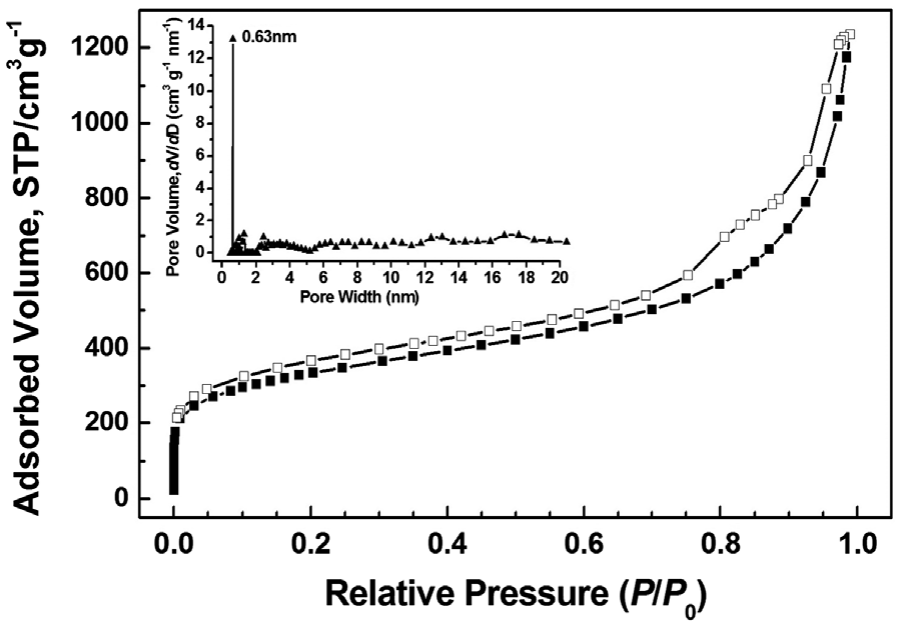
Nitrogen adsorption–desorption isotherms of **HCP-TCPP** measured at 77 K and related PSD profile (inset) calculated by NLDFT.

Porosity of the obtained polymer was studied by sorption analysis using nitrogen as the sorbate molecule. Nitrogen adsorption–desorption isotherms of polymer measured at 77 K are shown in Figure 5, which exhibit a combination of type I and II nitrogen sorption isotherms according to the IUPAC classification.[29] The increase in the nitrogen sorption at a high relative pressure above 0.9 may arise in part from interparticulate porosity associated with the meso-/macrostructures and interparticular voids of the sample.[30,31] Hysteresis can be observed apparently in the whole range of relative pressure based on the isotherms, which might be attributed to the swelling in a flexible polymer framework induced by adsorbate molecules dissolved in nominally nonporous parts of the polymer matrix after filling of open and accessible voids or the restricted access of adsorbate to the pores blocked by narrow openings.[32–34] The BET specific surface area value of **HCP-TCPP** is 1050 m^2^ g^−1^, which is calculated in the relative pressure (*P*/*P*_0_) range from 0.01 to 0.1 according to the previous reports.[35] The specific surface area value can be comparable to reported porphyrin-based porous polymers obtained by different coupling polymerization methods such as oxidative coupling reaction [22] and Sonogashira–Hagihara coupling reaction.[15] The PSD of the porous polymer was calculated from the adsorption branch of the isotherm with the NLDFT approach, the dominant pore size of polymer is centered at 0.63 nm.

**Figure 6.**
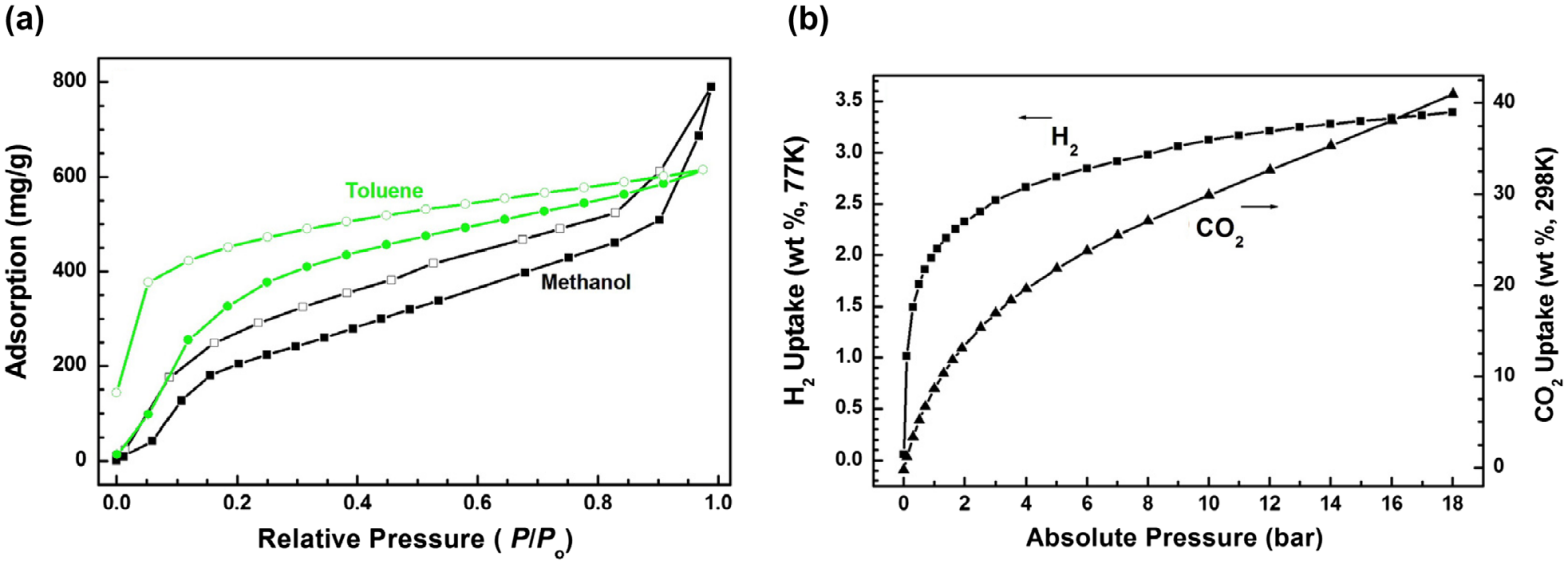
Vapor (a) and Gas (b) adsorption isotherms of **HCP-TCPP**.

As we know, the work related to vapor adsorption performance of hypercrosslinked porous polymers is very limited compared with gas adsorption. The porous polymers with high porosity allow potential access by a variety of small molecules.[36] Herein, we investigated the capability of the obtained material for adsorption of solvent vapors such as toluene and methanol. The related toluene and methanol adsorption–desorption isotherms at 298 K are shown in Figure 6(a). The adsorption amount of methanol by **HCP-TCPP** is high up to 800 mg g^−1^ (about 25.0 mmol g^−1^) at its saturated vapor pressure, which is higher than that of toluene (600 mg g^−1^, 6.5 mmol g^−1^) probably owing to the smaller molecular size for methanol. The high uptake capacity of **HCP-TCPP** for methanol could be ascribed to its high porosity and strong affinity to the absorbent molecules, which would have potential use to eliminate harmful small organic molecules in the environment. Further test also displays that polymer **HCP-TCPP**, possessing the high BET specific surface area and total pore volume, exhibits good hydrogen uptake of 3.44 wt % (77 K) and high carbon dioxide uptake of 41.1 wt % (298 K) at 18.0 bar (Figure 6(b)).

Porous polyporphyrin networks were reported to be the effective heterogeneous catalyst for a Knoevenagel reaction, which is a base-catalyzed condensation between aromatic aldehydes and acidic methylene derivatives.[37,38] Here, the Knoevenagel reaction between various aromatic aldehydes and malononitrile in the presence of **HCP-TCPP** was performed to study the catalytic activities of prepared porous polymer using reported optimal reaction conditions.[17] As shown in Table 1, aromatic aldehydes with different electron-withdrawing or electron-donating groups were all smoothly transformed to the corresponding desired products with good to excellent yields. Meanwhile, we also investigated the possibility of recycling **HCP-TCPP** in the model reaction using benzaldehyde and malononitrile as substrates. The catalyst was reused in the next round of condensation after being separated from the reaction mixtures by centrifugation, washed, and dried. It’s found that **HCP-TCPP** can also be reused in subsequent reactions without significant decrease in activity even up to 6 runs. No significant decrease in amount of catalyst after several times of reuse was observed.

**Table 1.** The Knoevenagel reaction between various aromatic aldehydes and malononitrile catalyzed by **HCP-TCPP**.


R	Catalyst	Yield (%)
Ph	no catalyst	5.3
Ph	**HCP-TCPP**	95
*p*-NO_2_Ph	**HCP-TCPP**	92
*o*-NO_2_Ph	**HCP-TCPP**	93
*p*-MePh	**HCP-TCPP**	85
*o*-MePh	**HCP-TCPP**	89
*p*-BrPh	**HCP-TCPP**	90
*o*-BrPh	**HCP-TCPP**	87
*p*-MeOPh	**HCP-TCPP**	88
*o*-MeOPh	**HCP-TCPP**	90
Ph	**HCP-TCPP** (2nd run)	95
Ph	**HCP-TCPP** (4st run)	94
Ph	**HCP-TCPP** (6st run)	92
Ph	**HCP-TCPP** (7st run)	89

## 4. Conclusions

To summary, hypercrosslinked porous polyporphyrin was obatined via the Friedel–Crafts alkylation of tetracarbazolylporphyrin using formaldehyde dimethyl acetal as an external cross-linker through metal-free protocol. The BET specific surface area value of **HCP-TCPP** is 1050 m^2^ g^−1^, which can be comparable to reported porphyrin-based porous polymers obtained by different coupling polymerization methods such as Suzuki coupling reaction and Sonogashira–Hagihara coupling reaction. The related gas adsorption capacities under high pressure for hydrogen and carbon dioxide have been investigated and the capabilities for adsorption of solvent vapors such as toluene and methanol have also been explored. The adsorption amount of methanol by **HCP-TCPP** is high up to 800 mg g^−1^ (about 25.0 mmol g^−1^) at its saturated vapor pressure, which is higher than that of toluene (600 mg g^−1^, 6.5 mmol g^−1^). Further test also displays that polymer **HCP-TCPP**, possessing the high BET specific surface area and total pore volume, exhibits good hydrogen uptake of 3.44 wt % (77 K) and high carbon dioxide uptake of 41.1 wt % (298 K) at 18.0 bar. Owing to the basic pyrrole containing macrocyclic cavities, the obtained porous polymer can also be used as a heterogeneous catalyst for the Knoevenagel condensation between various aldehydes and malononitrile. Aromatic aldehydes with different electron-withdrawing or electron-donating groups were all smoothly transformed to the corresponding desired products with good to excellent yields. It’s found that **HCP-TCPP** can also be reused in subsequent reactions without significant decrease in activity even up to 6 runs. No significant decrease in amount of catalyst after several times of reuse was observed.

## Disclosure statement

No potential conflict of interest was reported by the authors.

## Funding

This work was supported by the Science and Technology Foundation of Guizhou Province [grant number QKHJZ-2014–2176]; the Science and Technology Cooperation Project of Guizhou Province [grant number QKHLHZ-2015–7564]; the PhD Start-up Foundation of Zunyi Medical College [grant number F-710].
